# Changing landscapes, shifting reservoirs: land-use change and*Leptospira* ecology in Madagascar

**DOI:** 10.1128/aem.00487-26

**Published:** 2026-05-15

**Authors:** Nobuo Koizumi

**Affiliations:** 1 Department of Bacteriology I, National Institute of Infectious Diseases, Japan Institute for Health Security 739298 , Shinjuku, Tokyo, Japan; Michigan State University, East Lansing, Michigan, USA

**Keywords:** land-use change, *Leptospira*, leptospirosis

## Abstract

Land-use change is widely recognized as a driver of zoonotic disease emergence, yet its effects are often context-dependent. In an article published in *Applied and Environmental Microbiology*, J. A. Rajaonarivelo, K. M. Kauffman, T. M. Randriamoria, J. P. Herrera, et al. (92:e02061-25, 2026, https://doi.org/10.1128/aem.02061-25) show that shifts in small mammal communities across a disturbance gradient in Madagascar are associated with changes in *Leptospira* prevalence and lineage distribution. Introduced rodents contributed disproportionately to infections, whereas bats exhibited distinct patterns across habitat types. These findings highlight the importance of host community composition in shaping pathogen dynamics in changing landscapes.

## COMMENTARY

Human activities are transforming natural landscapes at an unprecedented pace. Across continents, particularly in tropical regions, forests are being converted into agricultural land, urban settlements, and other human-dominated environments. These changes alter biodiversity, host population density, and community composition, as well as human–animal contact patterns and host physiology. As a result, land-use change is widely recognized as a key driver of the emergence of zoonotic diseases (1–4). However, its effects are often complex and context-dependent, rather than following simple linear responses to disturbance, and can even lead to contrasting outcomes.

Among wildlife reservoirs, small mammals occupy an important position in zoonotic disease transmission. Rodents and bats are highly diverse, abundant, and capable of adapting to a wide range of habitats, including human-modified environments. Many zoonotic pathogens are maintained in these hosts, including viruses, bacteria, and protozoa that can spill over into human populations (5–8). One such pathogen is *Leptospira*, a genus of spirochete bacteria responsible for leptospirosis, a globally distributed zoonotic disease affecting both humans and animals (9). It is estimated that more than one million cases and 58,900 deaths occur annually worldwide, with 73% of cases reported in tropical regions (10). Pathogenic *Leptospira* species colonize the proximal renal tubules of reservoir hosts and are shed into the environment in urine, where they can persist under moist conditions and infect accidental hosts, including humans, via abraded skin or mucous membranes (11).

In an article published in *Applied and Environmental Microbiology*, Rajaonarivelo et al. investigate how land-use change shapes *Leptospira* ecology in Madagascar, a biodiversity hotspot undergoing rapid environmental transformation (12). By combining ecological surveys with molecular detection, the authors provide a detailed view of how host community structure and pathogen circulation vary across a gradient of anthropogenic disturbance.

A central finding of the study is the strong restructuring of host communities across land-use types. In terrestrial small mammals, endemic Malagasy species were primarily associated with forested habitats within the park, whereas non-native, introduced rodents and shrews dominated more disturbed, anthropogenic environments outside the park. These shifts in host community composition were accompanied by changes in *Leptospira* lineage distribution, suggesting a close link between host identity and pathogen diversity.

Patterns of infection further highlight the complexity of these relationships. In terrestrial small mammals, overall infection prevalence was relatively low (13.8%), yet the majority of infected individuals (86.1%) belonged to introduced species. Infection prevalence varied among host species and habitats, with no clear linear relationship with disturbance level. Binomial models further indicated that habitat type and year were the strongest predictors of infection. However, because localities were sampled sequentially rather than simultaneously, the effects of habitat and interannual variation could not be disentangled.

In contrast, bat communities exhibited a different pattern. While community composition differed among habitat types, overall diversity metrics remained relatively stable. Infection prevalence in bats was higher (37.7%) than in terrestrial small mammals but showed less variation across habitats. Consistent with this, habitat type was not a significant predictor of infection; instead, season emerged as the primary predictor, with higher odds during the hot dry season. However, because bats were only sampled in the park interior during this period—where prevalence was relatively high—the apparent seasonal effect may be confounded with spatial variation. These findings nevertheless suggest that highly mobile taxa, such as bats, may exhibit relatively stable transmission dynamics across environmental gradients, even as species composition shifts.

Phylogenetic analyses further revealed host-associated structuring of *Leptospira* lineages, with some lineages linked to non-native rodents and others restricted to native mammals, particularly bats. This finding underscores the importance of host identity in shaping pathogen diversity and suggests that land-use change may influence not only infection prevalence but also the ecological distribution of pathogen lineages. Notably, some lineages associated with non-native rodents were also detected in native species, raising the possibility of host switching events. Previous studies based on whole-genome sequencing have shown that certain *L. borgpetersenii* lineages are associated with dominant *Rattus* species in different regions, suggesting geographic structuring and host adaptation (13, 14). The patterns observed here may therefore reflect similar processes of host association and potential spillover across species boundaries. However, as lineage assignment in this study was based on single-gene sequencing, these interpretations should be considered with caution and validated by further studies.

Taken together, these results emphasize that the effects of land-use change on zoonotic pathogens are mediated primarily through changes in host community composition rather than overall biodiversity levels. In disturbed environments, the dominance of generalist, often non-native species may increase the relative contribution of highly competent reservoirs, while in more intact habitats, diverse native communities may support distinct pathogen assemblages. However, the absence of consistent linear trends with disturbance highlights the context-dependent nature of these processes.

These findings have important implications for understanding zoonotic disease risk. Leptospirosis is an environmentally transmitted disease, and human infection risk depends on both the presence of infected hosts and opportunities for exposure to contaminated environments. Anthropogenic landscapes, such as flooded rice fields in this study, can support high densities of reservoir hosts while facilitating human contact, and may therefore represent key interfaces for pathogen transmission. This study also reinforces the need to move beyond simple metrics of biodiversity when predicting disease risk. Instead, a focus on community composition, host competence, and ecological interactions is essential. The integration of ecological, microbiological, and molecular approaches, as demonstrated here, provides a powerful framework for disentangling these complex relationships.

As environmental change accelerates, understanding how different host communities respond to land-use transitions will be critical for anticipating shifts in pathogen transmission. Longitudinal studies, improved characterization of host competence, and the incorporation of environmental variables, such as hydrology, climate, and anthropogenic factors, including chemical pollution will further enhance our ability to predict disease risk (15). Ultimately, a One Health approach integrating human, animal, and environmental health will be essential for mitigating the impacts of zoonotic diseases in rapidly changing landscapes.

## References

[1] Faust CL, McCallum HI, Bloomfield LSP, Gottdenker NL, Gillespie TR, Torney CJ, Dobson AP, Plowright RK. 2018. Pathogen spillover during land conversion. Ecol Lett 21:471–483. doi:10.1111/ele.12904 29466832

[2] Guo F, Bonebrake TC, Gibson L. 2019. Land-use change alters host and vector communities and may elevate disease risk. Ecohealth 16:647–658. doi:10.1007/s10393-018-1336-3 29691680

[3] Carlson CJ, Brookson CB, Becker DJ, Cummings CA, Gibb R, Halliday FW, Heckley AM, Huang ZYX, Lavelle T, Robertson H, Vicente-Santos A, Weets CM, Poisot T. 2025. Pathogens and planetary change. Nat Rev Biodivers 1:32–49. doi:10.1038/s44358-024-00005-w 41789152 PMC7618822

[4] Rulli MC, D’Odorico P, Galli N, John RS, Muylaert RL, Santini M, Hayman DTS. 2025. Land use change and infectious disease emergence. Rev Geophys 63:e2022RG000785. doi:10.1029/2022RG000785

[5] Luis AD, Hayman DTS, O’Shea TJ, Cryan PM, Gilbert AT, Pulliam JRC, Mills JN, Timonin ME, Willis CKR, Cunningham AA, Fooks AR, Rupprecht CE, Wood JLN, Webb CT. 2013. A comparison of bats and rodents as reservoirs of zoonotic viruses: are bats special? Proc Biol Sci 280:20122753. doi:10.1098/rspb.2012.2753 23378666 PMC3574368

[6] Olival KJ, Hosseini PR, Zambrana-Torrelio C, Ross N, Bogich TL, Daszak P. 2017. Host and viral traits predict zoonotic spillover from mammals. Nature 546:646–650. doi:10.1038/nature22975 28636590 PMC5570460

[7] Strand TM, Lundkvist Å. 2019. Rat-borne diseases at the horizon. A systematic review on infectious agents carried by rats in Europe 1995-2016. Infect Ecol Epidemiol 9:1553461. doi:10.1080/20008686.2018.1553461 30834071 PMC6394330

[8] Ferreira ACR, Colocho RAB, Pereira CR, Veira TM, Gregorin R, Lage AP, Dorneles EMS. 2024. Zoonotic bacterial pathogens in bats samples around the world: a scoping review. Prev Vet Med 225:106135. doi:10.1016/j.prevetmed.2024.106135 38394962

[9] Giraud-Gatineau A, Nieves C, Harrison LB, Benaroudj N, Veyrier FJ, Picardeau M. 2024. Evolutionary insights into the emergence of virulent Leptospira spirochetes. PLoS Pathog 20:e1012161. doi:10.1371/journal.ppat.1012161 39018329 PMC11285912

[10] Costa F, Hagan JE, Calcagno J, Kane M, Torgerson P, Martinez-Silveira MS, Stein C, Abela-Ridder B, Ko AI. 2015. Global morbidity and mortality of leptospirosis: a systematic review. PLoS Negl Trop Dis 9:e0003898. doi:10.1371/journal.pntd.0003898 26379143 PMC4574773

[11] Bharti AR, Nally JE, Ricaldi JN, Matthias MA, Diaz MM, Lovett MA, Levett PN, Gilman RH, Willig MR, Gotuzzo E, Vinetz JM, Peru-United States Leptospirosis Consortium. 2003. Leptospirosis: a zoonotic disease of global importance. Lancet Infect Dis 3:757–771. doi:10.1016/s1473-3099(03)00830-2 14652202

[12] Rajaonarivelo JA, Kauffman KM, Randriamoria TM, Herrera JP, Wickenkamp N, Turpin M, Baudino F, Young HS, Soarimalala V, Goodman SM, Nunn CL, Tortosa P. 2026. Leptospira prevalence and lineages vary across land-use types due to shifts in small mammal communities. Appl Environ Microbiol 92:e02061-25. doi:10.1128/aem.02061-25 41615177 PMC12915484

[13] Koizumi N, Wada T, Morita M, Mu J-J, Ohnishi M. 2020. Comparative genomic analysis of Leptospira borgpetersenii serogroup Javanica isolated from Rattus species in Southern Japan, Philippines, and Taiwan. Infect Genet Evol 85:104447. doi:10.1016/j.meegid.2020.104447 32619638

[14] Koizumi N, Morita M, Nuradji H, Noor SM, Dharmayanti N, Randusari P, Mu J-J, Solante RM, Saito N, Ariyoshi K, Ha HTT, Wada T, Akeda Y, Miura K. 2022. Comparative genomic analysis of Leptospira spp. isolated from Rattus norvegicus in Indonesia. Infect Genet Evol 102:105306. doi:10.1016/j.meegid.2022.105306 35618255

[15] Mahon MB, Sack A, Aleuy OA, Barbera C, Brown E, Buelow H, Civitello DJ, Cohen JM, de Wit LA, Forstchen M, Halliday FW, Heffernan P, Knutie SA, Korotasz A, Larson JG, Rumschlag SL, Selland E, Shepack A, Vincent N, Rohr JR. 2024. A meta-analysis on global change drivers and the risk of infectious disease. Nature 629:830–836. doi:10.1038/s41586-024-07380-6 38720068

